# Estimating the Impact of COVID-19 Pandemic Related Lockdown on Utilization of Maternal and Perinatal Health Services in an Urban Neighborhood in Delhi, India

**DOI:** 10.3389/fgwh.2022.816969

**Published:** 2022-03-29

**Authors:** Bireshwar Sinha, Nonita Dudeja, Sarmila Mazumder, Tivendra Kumar, Priyanka Adhikary, Nivedita Roy, Temsunaro Rongsen Chandola, Rajesh Mehta, Neena Raina, Nita Bhandari

**Affiliations:** ^1^Centre for Health Research and Development, Society for Applied Studies, New Delhi, India; ^2^Department of Biotechnology (DBT)/Wellcome India Alliance, Hyderabad, India; ^3^Regional Office for South-East Asia, World Health Organization, New Delhi, India; ^4^Knowledge Integration and Translational Platform at Centre for Health Research and Development, Society for Applied Studies, New Delhi, India

**Keywords:** COVID-19, antenatal—postnatal, maternal health care utilization, perinatal care, primary care (MeSH)

## Abstract

**Objective:**

To estimate utilization of maternal, perinatal healthcare services after the lockdown was implemented in response to the COVID-19 pandemic compared to the period before.

**Methods:**

This study conducted in Dakshinpuri, an urban neighborhood in Delhi, reports data over a 13-month period which includes the period “before lockdown” i.e., October 1, 2019 to March 21, 2020 and “after lockdown” i.e., March 22 to November 5, 2020. The period “after lockdown” included the lockdown phase (March 22 to May 31, 2020) and unlock phase (June 1 to November 5, 2020). Mothers delivered during this period in the study area were interviewed using semi-structured questionnaires. In-depth interviews (IDIs) were conducted in a subsample to understand the experiences, challenges, and factors for underutilization of healthcare services.

**Findings:**

The survey covered a total population of 21,025 in 4,762 households; 199 eligible mothers (mean age 27.4 years) were interviewed. In women who delivered after lockdown against before lockdown, adjusted odds of having >2 antenatal care visits in the third trimester was 80% lower (aOR 0.2, 95% CI 0.1–0.5); proportion of institutional deliveries was lower (93 vs. 97%); exclusive breastfeeding during first 6 months of birth (64.5 vs. 75.7%) and health worker home visitation within 6 weeks of birth (median, 1 vs. 3 visits) were substantially lower. Fear of contracting COVID-19, poor quality of services, lack of transportation and financial constraints were key issues faced by mothers in accessing health care. More than three-fourth (81%) of the mothers reported feeling down, depressed or hopeless after lockdown. The major factors for stress during lockdown was financial reasons (70%), followed by health-related concerns.

**Conclusion:**

COVID-19 pandemic-related lockdown substantially affected maternal and perinatal healthcare utilization and service delivery.

## Introduction

The COVID-19 pandemic and the resultant nationwide lockdowns have adversely affected the delivery of essential maternal and perinatal services and their utilization across countries. The effect is reported to be tremendous in low- and middle-income countries where health systems are already overstretched due to high population density and poor resource availability (1, 2). Modeling studies have estimated that even a modest reduction of 10% in coverage of pregnancy and neonatal health care as an effect of the pandemic-related lockdowns may result in an additional 28,000 maternal deaths and 168,000 neonatal deaths globally (3, 4).

The maternal and child health indicators in India improved remarkably over a decade with an increase in institutional delivery rate from 38.7% in 2005–2006 to 78.9% in 2015–16 and a reduction in the infant mortality rate from 57 to 41 per 1,000 live births during the same period as a result of sustained efforts (5). These acquired health gains may suffer substantial losses if the resource-constrained health care delivery system fails to deliver essential services owing to the crippling effect of the COVID-19 pandemic. Reports from hospital-based studies suggest that COVID-19 related national lockdowns have affected outpatient as well as essential health service utilization (6). A study in Nepal in 2020, reported a 52.4% reduction in institutional deliveries during the 9.5 weeks of national lockdown compared with 12.5 weeks before lockdown (7). A study in four tertiary hospitals in western India reported a 66% reduction in the number of emergency obstetric visits, a significant increase in in-hospital mortality among pregnant women, late intrauterine fetal death and stillbirth, during the 10 weeks after lockdown compared to 10 weeks before (8). Estimating the effect of the COVID-19 pandemic on antenatal, intrapartum and postnatal service delivery and its utilization at the community level was deemed to be important.

In India, the nationwide lockdown was announced on 22nd March 2020 to curtail the transmission and prepare the health systems for an unprecedented rise in the number of COVID-19 cases. We conducted a community-based study to estimate utilization patterns of antenatal, intrapartum and postnatal care services in women who delivered after the lockdown was implemented (i.e., on or after 22nd March 2020) compared to those who delivered before lockdown. Additionally, we endeavored to estimate the out-of-pocket expenses and understand the challenges faced by women related to health care utilization after the lockdown was implemented and its possible solutions for such situations in future.

## Materials and Methods

### Study Design

This observational study was conducted in Dakshinpuri, an urban low-middle income neighborhood in South Delhi, India. The community survey was done from August 2020 to November 2020 and the information on utilization of antenatal, intrapartum and postnatal care services was collected from women who delivered between October 2019 to November 2020. This overall 13-month period includes the period “before lockdown” i.e., 1st October 2019 to 21st March 2020 and the period “after lockdown” was implemented i.e., 22 March to 5th November 2020. The period “after lockdown” included the lockdown phase (22nd March 2020 to 31st May 2020) and the unlock phase (1st June to 5th November 2020).

The primary study outcomes were the proportion of women who received the scheduled (at least 2) third-trimester antenatal care (ANC) visits and the proportion of institutional deliveries.

### Sample Size and Sampling

Assuming, 65% of pregnant women received scheduled ANC visits in the 3rd trimester and 85% had institutional deliveries (5), a 20% absolute reduction in the coverage due to lockdown, a minimum sample size of 95 women each who delivered before lockdown and after lockdown was required with 80% power and 5% alpha error. Considering a 20% refusal rate the total sample size deemed required was 230 women. Based on a birth rate of 15/1,000 population (9), around 16,000 population were required to be surveyed. Given the average family size of 4.5 in the study area, nearly 3,560 households had to be covered.

The study area in Dakshinpuri comprised 11 blocks of various size with a total population of ~1 lakh. Each of these blocks was assigned a number. The survey was done in the order in which blocks were randomly selected in STATA. The survey in one block was completed before moving to the next until the required sample size was achieved. A total of five complete blocks were surveyed.

### Ethics Statement

Approval was obtained from the Ethics Review Committee CHRD SAS, Delhi. Written informed consent was obtained from all participants before the interview. The study was registered in the Clinical trials registry India [CTRI/2020/07/026389].

### Data Collection

A door-to-door household survey was conducted. All households were visited in the selected blocks and population enumeration forms were filled. In households with women who delivered a baby on or after 1st October 2019, sociodemographic, antenatal, intrapartum and postnatal care details including out-of-pocket expenditure were captured using a semi-structured survey questionnaire that had been earlier pretested. Wherever possible, reported information was verified from available health records like Mother and Child Protection card, hospital records, and medical prescriptions. Women were also interviewed to understand the social and psychological impact of COVID-19.

In-depth interviews (IDI) were conducted by trained anthropologists in a subsample of 25 women who delivered after lockdown to understand their experiences, challenges, and barriers in accessing health care services. We also interviewed community health workers, government doctors and private health practitioners to get suggestions on possible solutions to combat challenges related to health care access and delivery in the event of similar outbreak-like situations in the future. Each interview lasted for 40–60 min.

### Data Analysis

Data analysis was done by using software STATA 16 (TX, USA). Antenatal, intrapartum and postnatal care service utilization outcomes were compared across the before lockdown (1st October 2019 to 21st March 2020) and after lockdown periods (22nd March 2020 to 5th November 2020) using the chi-square test for categorical outcomes and *t*-test (normal distribution) or Wilcoxon rank-sum test (non-normal distribution) for continuous outcomes. The month-wise proportions of women who had two or more antenatal care visits in the third trimester, and institutional deliveries (primary outcomes) were plotted using the time-series plots to study trends across before and after lockdown (including the lockdown and unlock phase). For categorical outcomes, we used generalized linear model (GLM) of the binomial family with log-link to estimate the adjusted Odds Ratio (aOR) and its 95% confidence intervals (95% CI) of poor utilization of health care services after lockdown against before lockdown (reference) adjusted for potential confounding variables viz. family income, religion, caste, education, and parity. For continuous outcomes, GLM for poisson distribution was used with an identity link. Catastrophic expenditure was defined as out-of-pocket expenditure for maternal and newborn healthcare services exceeding a threshold of 10% of the household annual income (10, 11).

The audio recordings of the in-depth interviews (IDIs) were expanded, reviewed and transcribed. Each transcript was analyzed using a framework analysis technique that employed sifting, charting and systematically sorting the data to allow key issues, themes and sub-themes to emerge. The data is then presented in the form of general concepts illustrated with verbatims.

## Results

**Figure 1:**
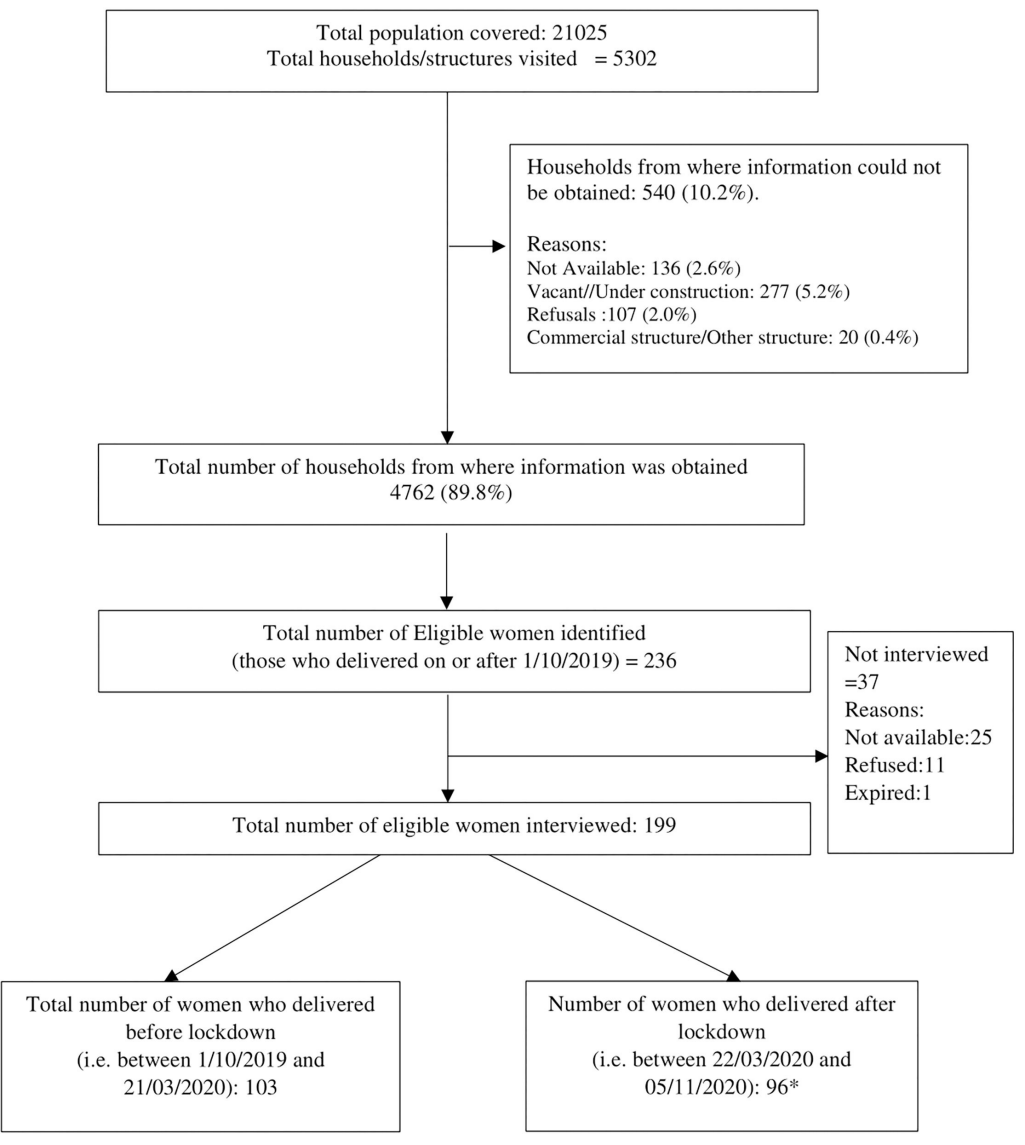
Flow of participants in the study. *Total number of women in which in-depth interviews performed for the qualitative component: 25.

The total population covered through a door-to-door survey was 21,025 in 4,762 households. Of the total 236 women who delivered a baby on or after 1st October 2019 identified, 199 (84%) were interviewed. Among them, 103 (52%) had delivered before lockdown and 96 (48%) had delivered after lockdown (Figure 1). Overall, the mean (SD) age of the women was 27.4 (4.1) years, 48% were primiparous, and 66% had 10 or more years of education. The sociodemographic profile of women who delivered before and after lockdown was similar (Table 1).

**Table 1:** Sociodemographic profile of study participants^a^.

**Variables**	**Delivered before lockdown** **(*N* = 103)** ***n* (%)**	**Delivered after lockdown** **(*N* = 96)** ***n* (%)**
Type of family: extended	40 (62.1)	56 (58.3)
Annual family income in INR^b^: median (IQR)	180,000 (120,000–240,000)	180,000 (120,000–252,000)
**Religion**		
Hindu	92 (89.3)	84 (87.5)
Muslim	10 (9.7)	10 (10.4)
Others	1 (0.9)	2 (2.0)
Family social class: general	28 (27.1)	29 (30.2)
Women's age in years: mean (SD)	27.9 (4.3)	26.7 (3.8)
**Parity**		
1	47 (45.6)	49 (51.0)
2	44 (42.7)	31 (32.3)
3 or more	12 (11.7)	16 (16.7)
Women's education: mean years of schooling (SD)	10.4 (4.0)	10.3 (3.6)
Women's occupation: homemaker	99 (96.1)	89 (92.7)

a *Figures indicate n (%) unless indicated otherwise*.

b *1 USD = 73.5 INR on 5th Nov 2020 (cbic.gov.in)*.

### Antenatal Care Services

**Figure 2:**
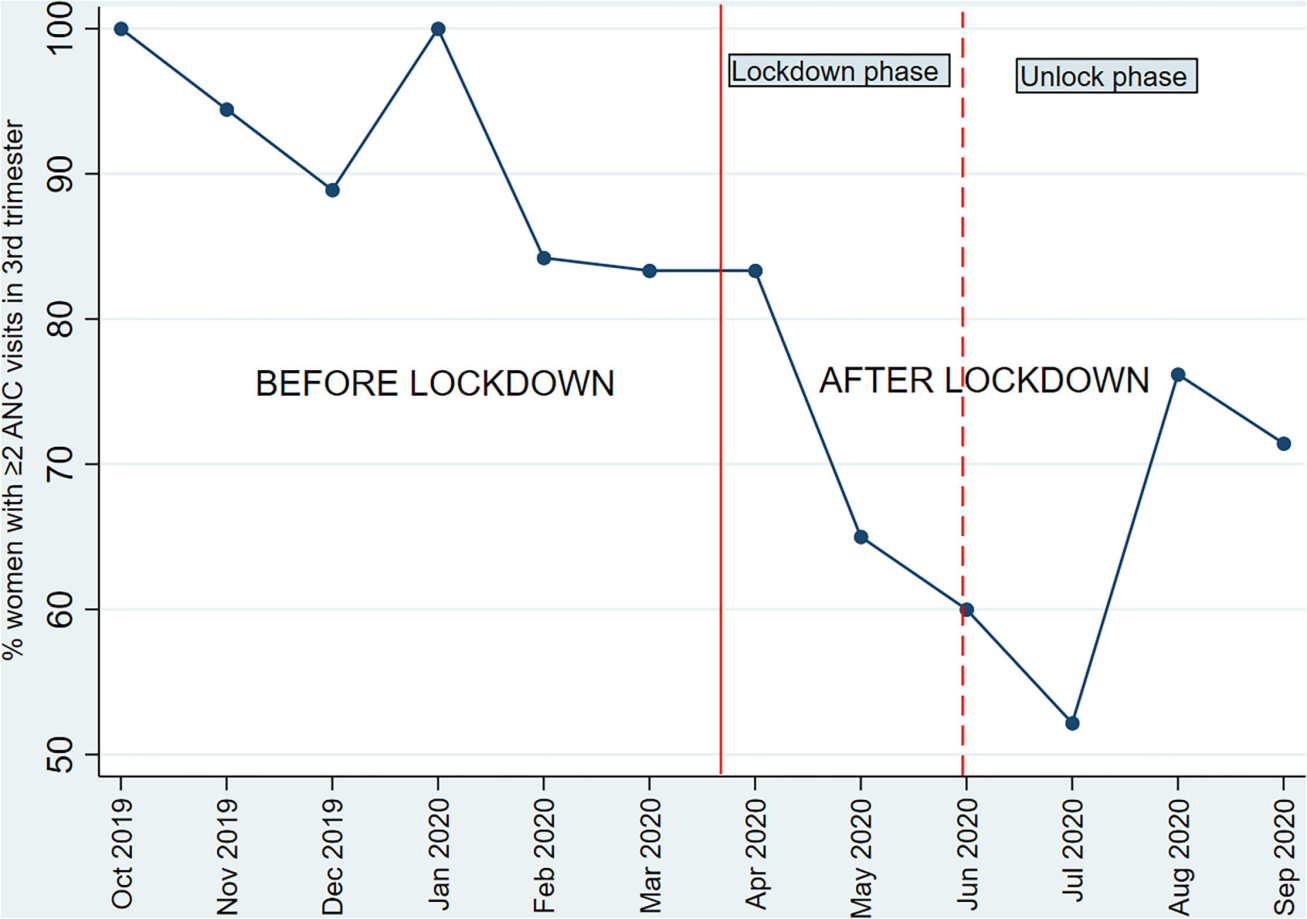
Month wise proportion of pregnant women who had ≥2 antenatal visits in the third trimester*. *Only one of the study women had delivered in October 2020 and was considered in the previous month for this analysis; the woman had ≥2 ANC visits in the 3rd trimester.

Information on ANC visits was available from ANC cards or health center records in 81.3% and through respondent's recall in 18.7%. The adjusted odds of having >2 ANC visits in the third trimester were 80% lower in women who delivered after lockdown against those who delivered before lockdown (aOR 0.2, 95% CI 0.1–0.5). Among the women who delivered after lockdown, 84% had ≥4 ANC visits compared to 97% among those who delivered before lockdown (aOR 0.3, 95% CI 0.1–0.5). The mean ANC visits were lower in women who delivered after lockdown than those who delivered before lockdown (adjusted mean difference −1.0, 95% CI −1.7 to −0.3) (Table 2). A drop in the utilization of ANC services was noted from April 2020 and was greatest during May to July 2020 (Figure 2).

**Table 2:** Health service utilization among mothers who delivered before and after lockdown^a^.

**Health utilization outcomes**	**Before lockdown** ***N* = 103** ***n* (%)**	**After lockdown** ***N* = 96** ***n* (%)**	**Unadjusted OR/mean difference** **(95%CI)**	**Adjusted^c^ OR/mean difference** **(95% CI)**
**Antenatal care**				
Women with 2 or more ANC visits in third trimester	94 (91.3)	65 (67.7)	0.2 (0.1–0.4)	0.2 (0.1–0.5)
Women with four or more total ANC visits	100 (97.3)	81 (84.3)	0.2 (0.1–0.6)	0.3 (0.1–0.5)
No. of ANC check-ups: mean (SD)	6.6 (1.8)	5.6 (2.1)	−0.9 (−1.7 to −0.3)^b^	−1.0 (−1.7 to −0.3)^b^
Women who received two tetanus injections	96 (93.2)	80 (83.3)	0.4 (0.1–0.9)	0.3 (0.1–0.9)
**Intrapartum care**				
Institutional delivery	100 (97.1)	90 (93.5)	0.5 (0.1–1.9)	0.5 (0.1–2.3)
**Place of institutional delivery**				
Government facility	57 (55.3)	49 (51.0)	0.9 (0.5–1.6)	0.8 (0.4–1.4)
Private facility	43 (41.7)	41 (42.7)	1.1 (0.6–2.0)	1.1 (0.6–2.0)
Delivery conducted by skilled personnel	100 (97.1)	91(94.8)	0.6 (0.1–2.3)	0.5 (0.1–2.3)
**Postnatal and newborn care**				
Discharged ≤ 48 h^d^	35 (35.0)	45 (50.0)	1.9 (1.1–3.3)	2.1 (1.2–4.0)
Newborn complications	27 (26.2)	27 (28.1)	1.1 (0.6–2.1)	1.1 (0.6–2.0)
Preterm	5 (4.9)	7 (7.3)	1.5 (0.5–5.0)	1.7 (0.5–5.9)
Low birth weight	19 (18.5)	17 (17.7)	0.9 (0.5–2.0)	0.9 (0.4–2.0)
Signs of possible serious bacterial infection (PSBI)	4 (3.8)	6 (6.3)	1.7 (0.4–6.0)	1.6 (0.4–5.8)
Jaundice	8 (7.7)	8 (8.3)	1.1 (0.4–3.0)	1.1 (0.4–3.2)
Delayed (>1 h) initiation of breastfeeding	50 (48.5)	48 (50.0)	1.1 (0.6–1.8)	1.0 (0.5–1.7)
Exclusive breastfeeding (during first 6 months of life)	78 (75.7)	62 (64.5)	0.6 (0.3–1.1)	0.6 (0.3–1.1)
HBNC^e^ care visit within 2 days of delivery	17 (16.5)	12 (12.5)	0.7 (0.3–1.6)	0.7 (0.3–1.6)
Any HBNC visits by ASHA/ANM within 6 weeks postdelivery	87 (84.5)	49 (51.0)	0.2 (0.1–0.4)	0.2 (0.1–0.4)
Number of HBNC visits within 6 weeks postdelivery: median (IQR)	3 (2–5)	1 (0–2)	−2.1 (−2.5 to −1.6)^b^	−2.1 (−2.6 to −1.7)^b^
Advice on use of postnatal contraceptive devices	62 (64.6)	37(35.9)	0.3 (0.2–0.6)	0.3 (0.2–0.5)
**Out-of-pocket health expenditure**				
Catastrophic expenditure for maternal and newborn care	31 (30.1)	33 (34.4)	1.2 (0.7–2.2)	1.3 (0.7–2.4)

a *Figures indicate n (%) unless indicated otherwise*.

b *β-coefficient or difference in means*.

c *OR, Odds ratio; Adjusted for family income, religion, caste, education, and parity*.

d *Among those who had institutional deliveries*.

e *HBNC, Home based newborn care; ASHA, Accredited social health worker; ANM, Auxilliary nurse-midwife*.

The most common barriers reported in availing ANC services by women who delivered after lockdown were poor quality of care (55%), lack of conveyance to access care (41%) and long waiting time (39%) (Supplementary Table 1).

IDIs revealed that 8/25 (32%) of the women had to access ANC services from private facilities at higher cost by borrowing money, exhausting bank savings or selling jewelery. Women also reported that they missed ANC check-ups and/or scheduled ultrasounds due to lockdown and lack of transport. There were also reports of poor quality of care and hesitancy of health staff in conducting physical examination (Table 3).

**Table 3:** Experiences and challenges faced by women in seeking maternal and perinatal services as reported in in-depth interviews.

**Challenges and barriers faced**	**Verbatim (English translated)**
Inadequate human resources and poor quality of care	“I missed my last ultrasound during my pregnancy. Nurses used to avoid coming close. Doctors weren't physically examining/touching. They used to observe from a distance, it was a very strange feeling. Nurses did not even talk properly.” “First of all, there was only one person who was managing the hospital billing counter section. The queues were long, and one hospital staff was trying to manage the queue. His behaviour was unprofessional.” “Two women were asked to lie down on a 2.5 feet narrow delivery table in labour room. I was one of them. I was very scared of falling. Moreover, the toilet in the labour room was very dirty. The floor was blood-stained and the toilet had a foul stinking smell of urine.”
Irregular contact with community health workers	“Anganwadi was closed during the lockdown. ASHA did not come to visit, neither did she contacted me over the phone. She called me only once in June after the lockdown was over. She asked about my wellbeing and told me to register my name for ANC once the dispensary opens. But for the past 3–4 months, I did not have any antenatal checkup. I did whatever I could manage at home.”
Unprofessional behavior	“It is difficult to explain in words what I have gone through during my pregnancy. I would not recommend others to go to that public hospital for delivery. Behavior of hospital staff was unprofessional; I was not allowed to see the doctor. They told me to come in after two days.”
Lack of transportation	“I didn't get an auto on time. Bus service was not operational. Due to this, I faced great difficulties during my pregnancy and at the time of delivery”. “My delivery happened at home; the baby had come out. I could not make it to the hospital as I could not arrange for a mode of transport on time.”
Financial constraints	“I had to spend more than two lakhs on my delivery. Had it not been for corona, I would have gone to the Government hospital. There was no need to go to any private hospital.” “In private hospitals, staff behavior is respectful, and in public hospitals there is a tendency to make us go round in circles. We went to a private hospital for delivery, but the charges were Rs. 30000 and the costs of medicines and bed were separate. We surrendered. We thought we will go back to the public even if we must deal with the difficulties; or else, from where will get 30000?” “For our child there are lots of expenses, which are difficult to bear after both my husband's and mother's jobs were lost. My breast milk is also not adequate because I am not able to have enough food.”
Fear of contracting COVID-19	“Because of COVID-19, we went to a private hospital (for delivery) but had a lot of trouble because to find a hospital that did not have COVID19 patients. Otherwise, we always used to go to a Government hospital because it is nearby my home.”
Social stigma associated with COVID-19	“We never went out as my daughter is very young. We never took her out because of so many cases of Corona infections. Recently, the neighboring lane was sealed. It has been only a week that the lane had opened. The entire family staying in front of us was COVID positive. We got so scared that neither did we go down nor let our children go down. We told the rest of the neighbors also to not go near them. Once the fear gets inside your head, you automatically tend to react this way, to stay away from the people having COVID, and not talk to them.”

### Intrapartum Care

**Figure 3:**
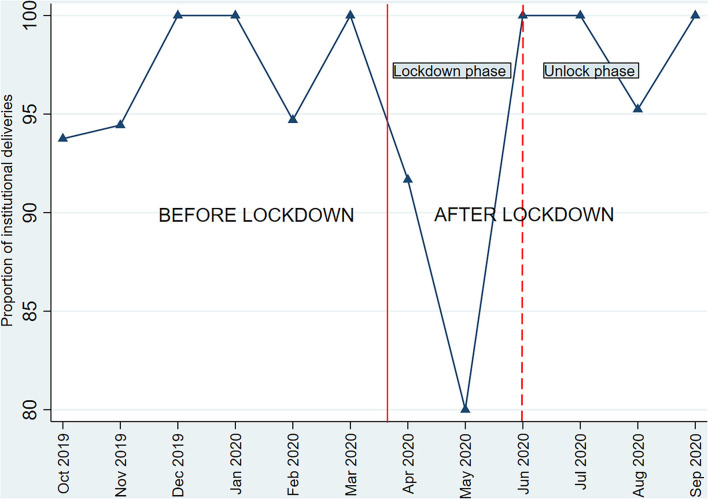
Month wise institutional delivery rates*. *Only one of the study women had delivered in October 2020 and was considered in the previous month for this analysis, the woman had an institutional delivery.

Among women who delivered after lockdown and before lockdown, the proportion of institutional deliveries was 93 and 97%, respectively (aOR 0.5, 95% CI 0.2–2.3). Further analysis showed that compared to before lockdown, the institutional delivery rate was substantially lower during the lockdown phase at 85% and seemed to improve during the unlock phase to 98% (*p* = 0.02) (Figure 3). We noted a slightly higher proportion of deliveries in private facilities after lockdown (42.7%) compared to before lockdown (41.7%) (Table 2).

In women who had home births, the major reason for not delivering in hospitals was the fear of COVID-19. More than one-fourth of women (28.1%) who delivered after lockdown reported issues related to intrapartum care. The key issues reported were fear of contracting COVID-19, poor quality of care and availability of no or few health staff (Supplementary Table 1).

IDIs revealed that one of the major barriers after lockdown was the shortage of hospital staff and doctors in the maternity care unit. Additionally, women reported challenges in finding a facility for delivery as many of the hospitals were converted into COVID-19 care centers. The women shared that their experiences in the labor room were distressing due to shortage of staff and unhygenic facilities. Non-availability of transport posed many challenges including not being able to reach the hospital in time for delivery (Table 3).

### Postnatal and Newborn Care

The proportion of women who were discharged ≤ 48 h of delivery was 50% after lockdown against 35% before lockdown (aOR 2.1, 95% CI 1.2–4.0). The proportion of newborns with any complications (preterm birth or low birthweight or possible serious bacterial infection or jaundice) was 28.1 vs. 26.2% after and before lockdown. Exclusive breastfeeding during the first 6 months of birth was 64.5% among infants born after lockdown compared with 75.7% in those born before lockdown (Table 2). The major reason for lower exclusive breastfeeding among infants born after lockdown, was fear of passing COVID-19 infection to the child and to avoid close contact (Supplementary Table 1). The odds of having home-based newborn care (HBNC) visit by a health worker within 6 weeks of delivery was 80% lower after lockdown against those before lockdown (aOR 0.2, 95% CI 0.1–0.4, Table 2). In 16/25 IDIs, the mothers reported that ASHA and Anganwadi workers were unavailable during the lockdown period for ANC or postnatal services for recently delivered women (Table 3).

### Out-Of-Pocket Expenditure

Out-of-pocket expenditure related to maternal and newborn care was reported by 91.7% of women who delivered after lockdown and 89.3% of those who delivered before lockdown; Catastrophic expenditure was incurred by 34.4% families of women who delivered after lockdown as against 30.1% before lockdown (Table 2). The median (IQR) out-of-pocket expenditure was INR 7,750 (2,900–20,000) after lockdown and INR 7,050 (2,600–16,725) before lockdown. The expenses related to investigations, transportation, admission fees were higher after lockdown compared to before lockdown; however, these differences were not statistically significant (Supplementary Table 2).

### Social and Psychological Impact

The key reasons for the apprehension related to COVID-19 disease as reported by the mothers were their concern for young children (86%) and elderly (77%) getting infected, unavailability of vaccines (41%) or treatment (38%) and risk of death (28%). Around half (48%) reported that a COVID-19 infected person is discriminated in the community and family is treated as a social outcast.

More than two-thirds (69%) of mothers reported disinterest or lack of pleasure in carrying out routine activities, more than three-fourths (81%) reported feeling down, depressed or hopeless after lockdown. The major factors for stress during lockdown was financial reasons (70%), followed by health-related concerns (12%; Supplementary Table 3).

## Discussion

Our study showed that delivery and utilization of antenatal care services and postnatal home-based newborn care have significantly reduced after lockdown in the study population compared with before. Institutional delivery rates were substantially lower in the lockdown phase. Postnatal HBNC visits were substantially lower after lockdown against before. The lower exclusive breastfeeding rates in women who delivered after lockdown seems to be related to the fear of passing COVID-19 infections in children. Catastrophic expenditure related to maternal and newborn care tended to be higher after lockdown than before. Fear of contracting COVID-19, poor quality of care, lack of conveyance, availability of health care personnel and financial constraints were some key issues faced by mothers in accessing health care after the lockdown was implemented. COVID-19 infection was reported to be discriminated in the community. A large proportion of the mothers reported depressive symptoms, financial and health concerns were the major factors for their stress.

In our study, the probability of selection bias is low because we interviewed all eligible women in contiguous households within the randomly selected community blocks. We used a questionnaire-based interview method to collect information which may have some possibility of recall or reporting bias. For the primary outcomes, 81% of the information obtained on antenatal care were based on health cards or hospital records and information on all institutional deliveries were documented. Therefore, we do not expect a major issue of recall bias. However, there still might be some possibility of information bias because of poor quality of health record documentation after lockdown, given the increased workload and staff shortage due to redistribution of staff in COVID-19 care. Overall, the results are likely to be reliable and representative of the target population given the relatively low risk of bias.

Results from our community-based study corroborate with some previous hospital-based studies conducted in India and other countries. A study in four tertiary hospitals in western India, showed a 43% reduction in hospitalization rates among pregnant women and a 66% reduction in emergency obstetric visits during the 10 weeks after lockdown compared to 10 weeks before lockdown (8). Another facility-based study in Nepal showed a sharp decline in institutional births after lockdown with an average weekly reduction of 7.4% during lockdown (7). Although we did not find any differences in high-risk birth outcomes across the study periods, the Nepal study found a 30% higher risk of preterm birth and 46% higher risk of institutional stillbirths in the lockdown period compared to before lockdown. The Nepal study also reported a 3.5% increase in delayed breastfeeding initiation in the lockdown period compared to before and poor utilization of health services due to lack of transport, fear of COVID-19 disease transmission and poor quality of care (7). Studies in the UK (12) and Italy (13) have documented a rise in depressive symptoms and anxiety disorders in expectant or postpartum mothers due to the COVID-19 pandemic and lockdown. Our study together with the previous studies provides credible evidence on the adverse effects of the COVID-19 pandemic and lockdown on utilization and delivery of maternal and perinatal health services, social and psychological health. But the improvements in institutional deliveries in the unlock phase compared to the lockdown phase as observed in our study indicate efforts to normalize essential health services.

Our community-based study used a mix of quantitative and qualitative method to provide robust estimates of the utilization patterns of antenatal, intrapartum, and postnatal care services in women who delivered after the lockdown compared to those before lockdown. Nonetheless, study has some limitations. It is not powered for the secondary outcomes (e.g., exclusive breastfeeding, catastrophic expenditure) leading to wide confidence intervals. Besides, the study was done in an urban low-socioeconomic neighborhood and the findings reported may not be generalizable to other settings.

## Conclusions and Future Implications

This study suggests that delivery and utilization of antenatal care services and postnatal care have been substantially affected due to the COVID-19 pandemic and related lockdown. The COVID-19 disease is also associated with social stigma and psychological implications in the community. The findings highlight the urgent need for improving accessibility, availability, and quality of essential maternal and newborn care services. Based on our interviews with the medical officers, health workers and women, certain measures may improve the access to essential health services in the event of similar situations in future. Political commitment and smart financing mechanisms are necessary to strengthen primary care facilities to provide 24^*^7 maternal and perinatal health emergency services with dedicated manpower and availability of medicines. Subsidized rates for emergency maternal and child services in the private sector through public-private partnerships can help to minimize the out-of-pocket expenses. Health worker scheduled home visits should be mandatory and may be driven by increased incentivization. Task shifting through peripheral health workers who may be trained to provide emergency care for mother and child closer to home is an option. Clear communication to inform the community regarding the place of availability of maternal and perinatal health services, the nature of precautions at health facilities to restrict the COVID-19 transmission is crucial. The possibility of setting up teleconsultation and mobile clinics for routine services and mental health counseling to address the societal fears and concerns may be explored.

Further research is needed to understand the long-term impact of the pandemic on women's health, delivery of maternal and perinatal health services closer to home, and mechanisms to improve health care utilization in this vulnerable group. Implementation research in vulnerable communities may help to understand the best measures required to create a resilient health system to cope with similar outbreak situations in future.

## Data Availability Statement

The raw data supporting the conclusions of this article will be made available by the authors, without undue reservation.

## Ethics Statement

The studies involving human participants were reviewed and approved by Ethics Review Committee, Center for Health Research and Development Society for Applied Studies, Delhi. The patients/participants provided their written informed consent to participate in this study.

## Author Contributions

BS: conceptualization and study design, training of team, verification of data, data analysis and interpretation, and writing-original draft. ND: data collection, verification of data, data analysis and interpretation, and writing-original draft. SM: conceptualization and study design, funding acquisition, training of team, and writing-review and editing. TK: training of team, data collection, and writing-review. PA and NRo: data collection and writing-review. TR: training team and writing-review. RM and NRa: supervision and writing-review and editing. NB: conceptualization, funding acquisition, supervision, and writing-review and editing. All authors have approved the final version of the manuscript.

## Funding

This work was supported by Department of Newborn, Child and Adolescent Health, Regional Office for South-East Asia, World Health Organization (PO Number 202559399).

## Author Disclaimer

The authors alone are responsible for the views expressed in this article and they do not necessarily represent the views, decisions, or policies of the World Health Organization.

## Conflict of Interest

The authors declare that the research was conducted in the absence of any commercial or financial relationships that could be construed as a potential conflict of interest.

## Publisher's Note

All claims expressed in this article are solely those of the authors and do not necessarily represent those of their affiliated organizations, or those of the publisher, the editors and the reviewers. Any product that may be evaluated in this article, or claim that may be made by its manufacturer, is not guaranteed or endorsed by the publisher.

## Abbreviations

COVID-19: Coronavirus-associated disease &#8211 2019
ANC: Antenatal care
IDI: In-depth-Interviews
HBNC: Home-based newborn care
INR: Indian rupees
aOR: adjusted odds ratio.
