# Supplementary material for: Estimating the Impact of COVID-19 Pandemic Related Lockdown on Utilization of Maternal and Perinatal Health Services in an Urban Neighborhood in Delhi, India
Source: Front Glob Womens Health. 2022 Mar 29;3:816969. doi: 10.3389/fgwh.2022.816969 (PMC9002136; doi:10.3389/fgwh.2022.816969)
Supplement: Supplementary file 1 [file Data_Sheet_1.pdf]

Online Supplemental File

Estimating the impact of COVID-19 pandemic related lockdown on utilization of maternal and perinatal health services in an urban neighbourhood in Delhi, India

Authors and Affiliations

Bireshwar Sinha, Nonita Dudeja, Sarmila Mazumder, Tivendra Kumar, Priyanka Adhikary, Nivedita Roy, Temsunaro Rongsen Chandola, Rajesh Mehta, Neena Raina, and Nita Bhandari

Contents:

Supplementary Table 1. Issues in seeking antenatal, intrapartum and postnatal care before and after lockdown

Supplementary Table 2. Out-of-pocket expenditure on maternal and newborn care before and after lockdown

Supplementary Table 3. Social and psychological issues in mothers related to COVID-19 infection and lockdown

Supplementary Table 1. Issues in seeking antenatal, intrapartum and postnatal care before and after lockdown [a]

Variables	Before Lockdown N=103 n(%)	After lockdown N=96 n(%)
**Antenatal care**		
Number of women who reported issues in availing ANC services	-	88 (91.6)
Issues reported in availing ANC services[b]		
Poor quality of care[c]	NA	48 (54.5)
Conveyance unavailable, difficult to access	NA	36 (40.9)
Long waiting time	NA	34 (38.6)
High consultation fee	NA	12 (13.6)
Fear of contracting COVID-19	NA	6 (6.8)
**Intrapartum care**		
Number of women who delivered at home	3 (2.9)	6 (6.2)
Reasons for home delivery[b]		
Fear of going to hospital due to COVID-19	NA	5 (83.3)
Financial reasons	1 (33.3)	0 (0.0)
Heard services were stopped due to pandemic	NA	1 (16.7)
No conveyance/No one to accompany	NA	2 (40.0)
Sent back and told to come when pains increase	1 (33.3)	1 (16.7)
Sudden onset of pain	1 (33.3)	0 (0.0)
Number of women who reported any issues in delivery facility (among those who had institutional delivery)	9 (8.7)	27 (28.1)
Issues faced in delivery facility[b]		
No or very few staff available	2 (22.2)	8 (29.6)
Long wait time/Overcrowding	8 (88.9)	18 (66.7)
Unprofessional behaviour of staff	8 (88.9)	15 (55.6)
Poor quality of care	0 (0.0)	11 (40.7)
Fear of contacting COVID-19	NA	7 (25.9)
**Post-delivery stay**		
Number of women who were discharged within 24 hours of delivery	8 (7.7)	10 (10.4)
Reasons for discharge before 24 hours[b]:		
Mother and baby fine, discharged early	6 (75.0)	5 (50.0)
Staff said unsafe to stay in hospital	NA	5 (50.0)
Difficult to go travel hospital daily	1 (12.5)	4 (40.0)
Unhygienic place / Food not available	-	2 (20.0)
Financial problem	1 (12.5)	1 (10.0)
Scared to stay long in hospital	0 (0)	4 (40.0)
**Newborn care**		
Number of women who had delayed breastfeeding initiation	50 (48.5)	48 (50.0)
Reasons for delayed breastfeeding initiation[b]		
Unaware	2 (4.0)	8 (16.7)
Baby separated	14 (28.0)	13 (27.2)
Caesarean section	20 (40.0)	15 (31.3)

Baby handed over to mother late	11 (22.0)	7 (14.6)
Baby sick	7 (14.0)	6 (12.5)
Mother sick	2 (4.0)	2 (4.1)
Others	3 (6.0)	6 (12.5)
Number of women did not practice exclusive breastfeeding	25 (24.3)	34 (35.4)
Reasons for non-exclusive breastfeeding[b]		
Fear of passing COVID-19 infection to child	NA	32 (94.1)
To avoid close contact with child	NA	32 (94.1)
Inadequate milk output	15 (60.0)	21 (61.7)
Family socio-cultural practices	3 (12.0)	9 (26.5)
Others	6 (7.7)	6 (9.7)

[a] Figures indicate n (%) unless indicated otherwise
[b] Multiple responses
[c] Includes not examined or no investigations or BP not measured, or weight not taken, or medicines not provided
[d] NA: Not applicable

Supplementary Table 2. Out-of-pocket expenditure on maternal and newborn care before and after lockdown[a]

Variables	Before Lockdown N=92	After Lockdown N=88	Wilcoxon rank sum test P value
Total out of pocket expenditure[b]			
Median (IQR)	7050 (2600 to 16725)	7750 (2900 to 20000)	
Mean (SD)	14353.0 (21542.6)	28534.5 (78120.8)	0.620
Consultation fee			
Median (IQR)	0 (0 to 25)	0 (0 to 0)	
Mean (SD)	435.9 (1539.7)	392.6 (1776.4)	0.821
Cost of Medicine			
Median (IQR)	0 (0 to 1100)	0 (0 to 1236)	
Mean (SD)	1336.3 (2588.4)	1283.2(2481.9)	0.623
Investigations			
Median (IQR)	0 (0 to 1500)	0 (0 to 1900)	
Mean (SD)	1368.5 (2805.6)	1450 (3019.4)	0.588
Admission Fee			
Median (IQR)	0 (0)	0 (0 to 0)	
Mean (SD)	629.5 (2177.7)	1593.2 (4832.0)	0.347
Transportation			
Median (IQR)	100 (0 to 500)	100 (0 to 1000)	
Mean (SD)	381.5 (795.6)	567.7 (1231.9)	0.301
Food cost			
Median (IQR)	0 (0)	0(0 to 350)	
Mean (SD)	197.8 (600.5)	192.6 (364.9)	0.296
Newborn vaccination			
Median (IQR)	500 (0 to 600)	200 (0 to 600)	
Mean (SD)	1056.5 (2893.7)	300 (338.3)	0.405
Others[c]			
Median (IQR)	1450 (0 to 7500)	1200 (0 to 10000)	
Mean (SD)	9739.5 (21879.5)	22990.3 (78505.1)	0.684

[a] All values are mentioned in Indian National Rupee (INR), 1USD= 73.5 INR on 5th Nov 2020 (cbic.gov.in)

[b]As the data on expenses is skewed, we have presented both median and means

[c]Others includes miscellaneous costs including ultrasound costs, operation theatre charges and informal charges taken by hospital caretakers/attendants.

Supplementary Table 3. Social and psychological issues in mothers related to COVID-19 infection and lockdown

Variables	N=199<br>n(%)
**Apprehension about COVID[a]**	
People die	56 (28.1)
No treatment available	77 (38.7)
No vaccine available	81 (40.7)
Older people at higher risk	153 (76.9)
Young children at higher risk	171 (85.9)
Take people forcibly to hospital	20 (10.1)
**Perception about COVID in community [a]**	
Discriminated/ labelled in society/ family is treated as social outcast	95 (47.7)
People hide infection or do not seek help for fear of infection	69 (34.7)
People are scared because they are forcefully taken to hospital	45 (22.6)
Family members cannot meet the person in hospital	89 (44.7)
Heard that conditions in hospital are very poor	76 (38.2)
The family cannot go out to buy necessary requirements to survive	108 (54.3)
People don't come back alive or being taken away to hospitals to die	14 (7.0)
**Psychological issues during lockdown [a]**	
Feel disinterested or lack of pleasure in carrying out routine activities	139 (69.9)
Feel down, depressed, or hopeless	161 (80.9)
Trouble in falling/staying asleep or sleeping too much	122 (61.3)
Feel very tired or lack of energy	151 (75.9)
Poor appetite or eating too much	92 (46.2)
Trouble in concentrating, such as reading newspaper or watching TV	87 (43.7)
Moving or speaking so slowly that other people could have noticed or the opposite; being fidgety or restless	8 (4.0)
Feel dizzy, lightheaded, or faint when they listen to news about coronavirus	81 (40.7)
Thoughts of and/or caused self-harm	5 (2.5)
Feel paralyzed or suicidal ideation on being exposed to information about coronavirus	4 (2.0)
**Greatest stress due to COVID outbreak in participant mothers[a]**	
Health concerns	23 (11.6)
Financial concerns	139 (69.9)
Impact on children	11 (5.5)
Impact on family members	5 (2.5)
Impact on community	2 (1.0)
Access to food / groceries	4 (2.0)
Access to baby supplies	3 (1.5)
Not stressed	12 (6.0)

[a]Multiple responses
